# Rapid Diagnostic Tests for Malaria Diagnosis in the Peruvian Amazon: Impact of *pfhrp2* Gene Deletions and Cross-Reactions

**DOI:** 10.1371/journal.pone.0043094

**Published:** 2012-08-28

**Authors:** Jessica Maltha, Dionicia Gamboa, Jorge Bendezu, Luis Sanchez, Lieselotte Cnops, Philippe Gillet, Jan Jacobs

**Affiliations:** 1 Department of Clinical Sciences, Institute of Tropical Medicine, Antwerp, Belgium; 2 Instituto de Medicina Tropical “Alexander von Humboldt”, Universidad Peruana Cayetano Heredia, Lima, Peru; 3 Departamento de Ciencias Celulares y Moleculares, Facultad de Ciencias y Filosofia, Universidad Peruana Cayetano Heredia, Lima, Peru; Université Pierre et Marie Curie, France

## Abstract

**Background:**

In the Peruvian Amazon, *Plasmodium falciparum* and *Plasmodium vivax* malaria are endemic in rural areas, where microscopy is not available. Malaria rapid diagnostic tests (RDTs) provide quick and accurate diagnosis. However, *pfhrp2* gene deletions may limit the use of histidine-rich protein-2 (PfHRP2) detecting RDTs. Further, cross-reactions of *P. falciparum* with *P. vivax-*specific test lines and vice versa may impair diagnostic specificity.

**Methods:**

Thirteen RDT products were evaluated on 179 prospectively collected malaria positive samples. Species diagnosis was performed by microscopy and confirmed by PCR. *Pfhrp2* gene deletions were assessed by PCR.

**Results:**

Sensitivity for *P. falciparum* diagnosis was lower for PfHRP2 compared to *P. falciparum*-specific *Plasmodium* lactate dehydrogenase (Pf-pLDH)- detecting RDTs (71.6% vs. 98.7%, *p*<0.001). Most (19/21) false negative PfHRP2 results were associated with *pfhrp2* gene deletions (25.7% of 74 *P. falciparum* samples). Diagnostic sensitivity for *P. vivax* (101 samples) was excellent, except for two products. In 10/12 *P. vivax*-detecting RDT products, cross-reactions with the PfHRP2 or Pf-pLDH line occurred at a median frequency of 2.5% (range 0%–10.9%) of *P. vivax* samples assessed. In two RDT products, two and one *P. falciparum* samples respectively cross-reacted with the Pv-pLDH line. Two Pf-pLDH/pan-pLDH-detecting RDTs showed excellent sensitivity with few (1.0%) cross-reactions but showed faint Pf-pLDH lines in 24.7% and 38.9% of *P. falciparum* samples.

**Conclusion:**

PfHRP2-detecting RDTs are not suitable in the Peruvian Amazon due to *pfhrp2* gene deletions. Two Pf-pLDH-detecting RDTs performed excellently and are promising RDTs for this region although faint test lines are of concern.

## Introduction

In Peru, malaria is mainly endemic in the Amazon region, where it is the primary cause of morbidity in adults and the fourth in children [1]. According to the recommendations of the World Health Organization (WHO), diagnosis and treatment should be based on parasitological confirmation by either microscopy or malaria rapid diagnostic tests (RDTs) [2]. In Peru, most cases occur in rural areas where no microscopy is available. Currently, thick blood films (TBFs) of malaria suspected patients are sent for analysis to the most nearby health center, but this process takes several days and patients are often treated presumptively [3]. In such conditions RDTs could be useful, providing quick and accurate diagnosis, thereby leading to timely and correct treatment and reducing the severity and economic burden of disease. Besides, use of RDTs in the Peruvian Amazon has been demonstrated to be cost-effective [4].

RDTs are handheld cassettes detecting malaria parasites by an antigen-antibody reaction on a nitrocellulose strip which become visible as blue or cherry-red test lines. There are several detection antibodies, directed to different antigens: histidine-rich protein-2 (PfHRP2) and *Plasmodium falciparum*-specific *Plasmodium* lactate dehydrogenase (Pf-pLDH) for *P. falciparum*; *Plasmodium vivax*-specific pLDH (Pv-pLDH) for *P. vivax*, and pan-pLDH and aldolase which are common to all four *Plasmodium* species.

The occurrence of both *P. vivax* and *P. falciparum* in Peru requires an RDT type that detects and differentiates between both species as they require different treatment [2]. However, cross-reactions may occur, *i.e.* the presence of a visible *P. falciparum* test line among *P. vivax* samples and vice versa [5], [6], due to genuine antigen-antibody interactions or non-specific bindings [7]. In addition, *P. falciparum* parasites lacking the *pfhrp2* and *pfhrp3* genes, -encoding PfHRP2 and the related protein PfHRP3 respectively- have been recently described in Peru [8] indicating that the use of PfHRP2 detecting RDTs may be limited [8]. Previous evaluations of two PfHRP2 detecting RDTs in Peru demonstrated sensitivity for *P. falciparum* diagnosis of 95% [9] and 53.5% [10].

The aims of the present study were to assess diagnostic accuracy of a panel of different RDT products for malaria diagnosis in the Peruvian Amazon, with particular focus on the impact of *pfhrp2* and *pfhrp3* gene deletions on diagnostic sensitivity and of cross-reactions on diagnostic specificity.

## Methods

### Ethics statement

The study was approved by the Ethical Review Board of the Universidad Peruana Cayetano Heredia, Lima, Peru (Code SIDISI: 55587 and 55239). All patients with a positive TBF, performed as part of routine patient care, were included after signing informed consent. Written informed consent was obtained from the patient himself in the case of adults or from the parent/guardian in case of a minor (<18 years).

### Study site and population

**Figure 1:**
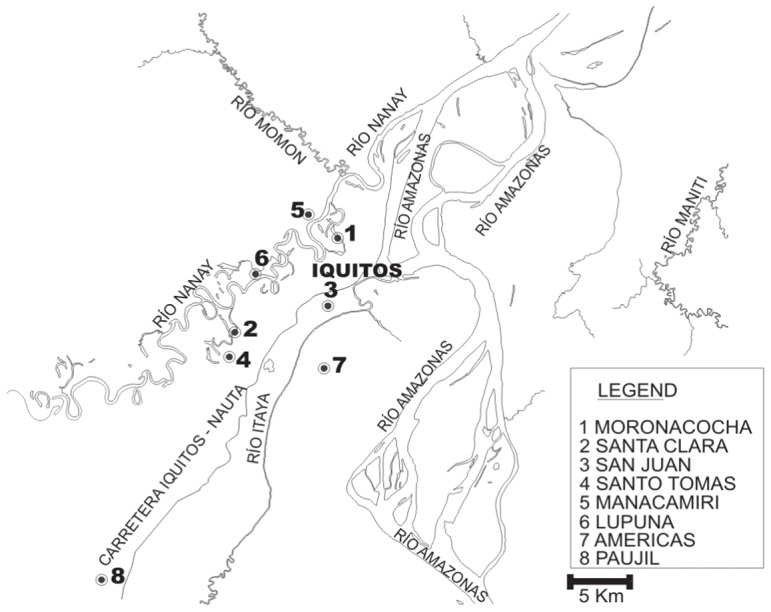
Map of included health centers. The village of Atalaya (−3.58, −73.75), located 59 km to the West of Iquitos, is not displayed on the map.

Several health centers around Iquitos (Figure 1) were included. Malaria in the Peruvian Amazon is perennial with a peak during the rainy season (November – May) and an incidence of 10–50 malaria cases per 1000 inhabitants per year [11]. Patients were included by either passive case detection (symptomatic patients presenting at the health centers) or active case detection (outreach teams performing malaria screening in epidemic communities). All patients with a positive TBF were included after signing informed consent. Previous antimalarial treatment, symptoms and travel history were recorded.

### Samples

EDTA anti-coagulated venous blood samples were drawn and transported to the laboratory of San Juan where RDTs were performed. After RDT performance, samples were aliquoted and stored at −20°C, usually within 24 hours (range 2–72 hours) after sample collection, pending further analysis.

### Malaria rapid diagnostic tests

Thirteen RDT products detecting several target antigens were selected (Table 1), based on good performance as documented by the WHO/Foundation for Innovative New Diagnostics (FIND) malaria RDT evaluation program [12], [13] or recent release on the market.

**Table 1:** Overview of RDT products and their lot numbers.

Product name	Manufacturer/distributor	Further referred to as	Target antigen Pf	Target antigen pan/Pv	Lot numbers	Recommended storage temperature
ADVANTAGE Mal Card	J. Mitra & Co., New Dehli, India	Advantage	Pf-pLDH	pan-pLDH	ACM171110	4–30°C
AZOG Malaria Pf/Pv	AZOG, Inc. New Jersey, USA	AZOG	PfHRP2	Pv-pLDH	58LAB017	2–30°C
CareStart™ Malaria Pf-pLDH/pLDH (Pf/PAN) Combo	Access Bio, Inc. New Jersey, USA	CareStart pLDH	Pf-pLDH	pan-pLDH	A10IL	4–30°C
CareStart™ Malaria HRP2/Pv-pLDH (Pf/Pv) Combo	Access Bio, Inc. New Jersey, USA	CareStart Pf/Pv	PfHRP2	Pv-pLDH	J10IV	4–30°C
Falcivax Rapid Test for Malaria Pv/Pf	Zephyr Biomedicals, Verna, India	Falcivax	PfHRP2	Pv-pLDH	81098	4–30°C
First Response Ag malaria pLDH/HRP2 combo test	Premier Medical Corporation Daman, India	First Response	PfHRP2	pan-pLDH	69I0610	4–30°C
Onsite Pf/Pv Ag rapid test	CTK Biotech, Inc. San Diego, USA	Onsite	PfHRP2	Pv-pLDH	F0810G2	2–30°C
PARACHECK Pf® (device)	Orchid Biomedical Systems Verna, India	Paracheck	PfHRP2	-	31795, 31797	4–45°C
Parascreen Rapid Test for Malaria Pan/Pf	Zephyr Biomedicals, Verna, India	Parascreen	PfHRP2	pan-pLDH	101176	4–30°C
SD Bioline Malaria Antigen test	Standard diagnostic, Hagal-dong, Korea	SDFK40	Pf-pLDH	pan-pLDH	MLRDT1001, MLRDT1002	1–40°C
SD Bioline Malaria Antigen P.f/pan	Standard diagnostic, Hagal-dong, Korea	SDFK60	PfHRP2	pan-pLDH	90026, 90017, 90096	1–40°C
SD Bioline Malaria Antigen P.f/P.v	Standard diagnostic, Hagal-dong, Korea	SDFK80	PfHRP2	Pv-pLDH	145015, 145016	1–40°C
SD Bioline Malaria Antigen P.f	Standard diagnostic, Hagal-dong, Korea	SDFK90	PfHRP2 and Pf-pLDH*	-	MFRDT1001, MFRDT1002	1–40°C

* SDFK90 contains 2 test lines specific for *P. falciparum*.

Both SDFK90 and Paracheck detect only *P. falciparum* and were included for evaluation of *P. falciparum* diagnosis. SDFK90 was only performed on *P. falciparum* samples and mixed infections. RDTs were purchased at the Institute of Tropical Medicine (ITM), Belgium and shipped to Peru. For logistic reasons (delays of delivery and shipment), some RDTs had to be performed on stored samples, in these cases median period of sample storage was 51 days (range 29–131 days).

### Test procedures

RDTs were performed according to the manufacturer's instructions except that the supplied transfer device was replaced by a micropipette. The first observer read test results within the specified reading time, the second and, when available, third observer within 10 additional minutes. Observers were blinded to each other's readings. In case of absence of the control line the test was repeated. A scoring system of five categories was used to assess line intensities [14]. Test results were based on consensus agreement in case of three observers. In all other cases, the result of the first observer was considered.

### Microscopy

At the laboratory of San Juan, species and parasite density were determined by TBF microscopy, assuming a white blood cell count of 8,000/µl [15]. For quality control (QC), 20% randomly selected slides, including those with interpretive problems, discordant RDT results, negative slides and suspected mixed infections were reexamined by two blinded expert microscopists at ITM. For parasite density the results of the first microscopist were considered except when QC indicated a density of more than two fold difference with the original count, in such cases mean of the two QC readings was considered.

### DNA extraction

DNA was extracted from 200 µl whole blood using QIAamp DNA blood Mini kit (QIAGEN, Venlo, The Netherlands), according to the manufacturer's instructions except for a dilution in 100 µl instead of 200 µl elution buffer.

### Species-specific PCR

In case of discordances between RDT and microscopy or between initial and QC microscopy, real-time PCR (*P. falciparum/P. vivax*) was performed [16] which was considered conclusive.

### Assessment of *pfhrp2* and *pfhrp3* gene deletions

Confirmed *P. falciparum* samples were assessed for *pfhrp2* and *pfhrp3* gene deletions by conventional PCR using primers and conditions as described elsewhere [8], [17]. For *pfhrp2*, two amplifications were performed: one of entire exon 2 (encoding PfHRP2) and another across exon 1 and exon 2 (exon1–2). Samples were considered lacking the *pfhrp2* gene when both amplifications failed to generate a PCR product. For *pfhrp3*, a single amplification of entire exon 2 was performed.

### PfHRP2 ELISA

The presence of PfHRP2 protein in whole blood samples was determined by enzyme linked immune sorbent assay (ELISA, Standard Diagnostic, Hagal-Dong, Korea) according to the manufacturer's instructions. ELISA was performed in all samples with *P. falciparum* infection, mixed infections and in *P. vivax* samples generating visible PfHRP2 lines.

### Statistical analysis

Diagnostic sensitivity (calculated with 95% confidence intervals (C.I.)) of the RDT products was defined as the number of *P. falciparum* or *P. vivax* samples with a visible *P. falciparum*-specific or Pv-/pan-pLDH test line respectively (regardless of the presence of another test line), divided by the total number of *P. falciparum* or *P. vivax* samples respectively. Mixed infections were not included for calculation. Cross-reactions were defined as *P. falciparum* samples generating a visible Pv-pLDH line or *P. vivax* samples generating a visible PfHRP2 or Pf-pLDH line.

Proportions were assessed for statistical significance using the Chi-square test or, in case of small sample size, the Fisher-exact test. A *p*-value<0.05 was considered significant.

Interobserver agreement was determined by kappa values (κ) for positive and negative readings and line intensity readings between the first pair of observers.

### Additional analysis

All microscopically confirmed *P. falciparum* samples that did not show a visible PfHRP2 line in more than one RDT product were repeated two times with all PfHRP2-detecting RDTs.

## Results

### Patients and samples

From December 2010–July 2011, 182 patients were included, in three patients malaria was not confirmed by microscopy nor by PCR. Final sample collection consisted of *P. falciparum* (n = 74), *P. vivax* (n = 101) and four mixed infections. The collected samples comprised 5% of all *P. falciparum* and *P. vivax* infections reported in Loreto region in that time period [1], [18]. Data of demography and parasite density are shown in Table 2. Nineteen patients, including the two asymptomatic cases, were included through active case detection performed once in Tarapoto (n = 5) and once in Atalaya (n = 14).

**Table 2:** Patient data and parasite density of the final sample collection.

	*P. falciparum* (n = 74)	*P. vivax* (n = 101)	Mixed infection (n = 4)
Sample collection period	Dec 2010–Jul 2011	Dec 2010–Mar 2011	Dec 2010–Mar 2011
Male gender	41 (55.4%)	52 (51.5%)	4 (100%)
Age, median years (range)	27.5 (4–74)	29 (2–76)	31.5 (4–47)
Children <15 years, number (%)	16 (21.6%)	24 (23.8%)	1 (25%)
Median parasite density/µl (range)	4,971.5 (0–78,208)	5,080 (255–58,880)	9,527.5 (5,204–22,321)
Asymptomatic patients (number)	1 (1.4%)	1 (1.0%)	0 (0.0%)
Antimalarial treatment past 2 weeks	4 (5.4%)*	0 (0.0%)	0 (0.0%)

* artesunate + mefloquine since 2 days (n = 1), chloroquine since 2 days (n = 2), full course of chloroquine/primaquine (n = 1) at least >1 week ago (exact date not known).

### Diagnostic sensitivity of the RDT products

PfHRP2-detecting RDTs had significantly lower sensitivity for *P. falciparum* diagnosis compared to Pf-pLDH-detecting RDTs (*p*<0.0001, Table 3), due to a subset of samples that consequently failed to generate a PfHRP2 line in all PfHRP2 RDT products tested, see results below.

**Table 3:** Sensitivity, faint line intensity and cross-reactions of the different RDT products for detection of *P. falciparum* and *P. vivax*.

	% Sensitivity (95% C.I.)		% of positive test lines with faint intensity*		Number of cross-reactions (%)	
RDT product	*P. falciparum* (n = 74)	*P. vivax* (n = 101)	PfHRP2/Pf-pLDH	Pv-/pan-pLDH†	*P. vivax* with PfHRP2/Pf-pLDH test line	*P. falciparum* with Pv-pLDH test line
*PfHRP2-detecting RDT*						
Paracheck	70.3 (58.5–80.3)	-	5.8	-	0 (0.0)	-
*PfHRP2 and pan-pLDH detecting RDTs*						
First Response	71.6 (60.0–81.5)	100.0 (94.6–100.0)	3.8	2.0	3 (3.0)	-
Parascreen	71.6 (60.0–81.5)	89.1 (81.4–94.4)	1.9	21.1	7 (6.9)	-
SDFK60	71.6 (60.0–81.5)	100.0 (94.6–100.0)	7.1	4.0	5 (5.0)	-
*PfHRP2and Pv-pLDH detecting RDTs*						
AZOG	71.6 (60.0–81.5)	87.1 (79.0–93.0)	17.0	79.5	2 (2.0)	0 (0.0)
CareStart Pf/Pv	71.6 (60.0–81.5)	100.0 (94.6–100.0)	5.7	10.9	11 (10.9)	0 (0.0)
Falcivax	71.6 (60.0–81.5)	100.0 (94.6–100.0)	1.9	7.9	5 (5.0)	0 (0.0)
Onsite	71.6 (60.0–81.5)	100.0 (94.6–100.0)	5.7	4.0	0 (0.0)	2 (2.7)
SDFK80	71.6 (60.0–81.5)	100.0 (94.6–100.0)	1.9	0.0	1 (1.0)	1 (1.4)
*Pf-pLDH and pan-pLDH detecting RDTs*						
Advantage	98.7 (92.7–100.0)	100.0 (94.6–100.0)	24.7	4.0	1 (1.0)	-
CareStart pLDH	98.7 (92.7–100.0)	99.0 (94.6–100.0)	9.6	8.0	10 (9.9)	-
SDFK40	97.3 (90.6–99.7)	100.0 (94.6–100.0)	38.9	1.0	1 (1.0)	-
*PfHRP2 and Pf-pLDH detecting RDT*						
SDFK90 PfHRP2 line	71.6 (60.0–81.5)	-	1.9	-	-	-
SDFK90 Pf-pLDH line	98.7 (92.7–100.0)	-	40.5	-	-	-

* cross-reactions excluded.

† only *P. vivax* samples were considered.

For *P. vivax* diagnosis, most RDTs performed equally well, except for AZOG (detecting Pv-pLDH) and Parascreen (detecting pan-pLDH) (Table 3), which failed to detect *P. vivax* samples at a median parasite density of 1,075/µl (range 255–4,532/µl) and 600.5/µl (range 255–10,720/µl) respectively.

The mixed infections were detected by all RDT products except for AZOG which displayed a single PfHRP2 line for a sample consisting predominantly of *P. falciparum* parasites.

For *P. falciparum*, faint test line intensities occurred more frequently among Pf-pLDH compared to PfHRP2-detecting RDTs (*p*<0.001, Table 3). For *P. vivax*, no overall difference in proportion of faint test lines was observed between Pv-pLDH versus pan-pLDH-detecting RDTs.

### Failure of *P. falciparum* diagnosis by PfHRP2-detecting RDTs and *pfhrp2* gene deletions

All PfHRP2-detecting RDTs failed to diagnose 21 *P. falciparum* samples (Table 4), whereas the Pf-pLDH-detecting RDTs detected all of them. Most samples (19/21) were lacking *pfhrp2* (no amplification of exon1–2 and exon2). The remaining two samples (PI151 and PI156) generated PCR products for *pfhrp2* exon1–2 and exon2. *Pfhrp2* gene deletions occurred at both low and high parasite densities (Table 4) and all patients were symptomatic. PfHRP2 ELISA of the 21 samples confirmed the absence of PfHRP2, with only one sample (PI26) showing a weak positive result (optical density ten-fold lower than other ELISA positive samples).

**Table 4:** *P. falciparum* samples not detected by PfHRP2-detecting RDTs: *pfhrp2* and *pfhrp3* PCR results and PfHRP2 ELISA results.

Sample and patient information							
Sample number	Sex	Age	Parasite density (/µl)	*pfhrp2* exon 1–2	*pfhrp2* exon 2	*pfhrp3* exon 2	PfHRP2 ELISA
PI138	f	56	0*	−	−	−	−
PI139	m	6	79	−	−	−	−
PI137	m	41	80	−	−	−	−
PI136	m	53	270	−	−	−	−
PI 24	f	20	752	−	−	−	−
PI113	f	30	876	−	−	−	−
PI142	m	21	1,000	−	−	−	−
PI151	m	28	1,222	+	+	−	−
PI 18	f	12	1,400	−	−	−	−
PI 78	m	37	2,808	−	−	−	−
PI156	f	36	3,480	+	+	+	−
PI135	m	7	4,784	−	−	−	−
PI153	m	70	5,080	−	−	−	−
PI140	f	20	5,640	−	−	−	−
PI 26	m	48	7,227	−	−	−	+/−
PI 27	f	67	7,840	−	−	−	−
PI163	m	65	16,552	−	−	−	−
PI 81	m	46	18,800	−	−	−	−
PI148	f	34	19,600	−	−	−	−
PI 74	m	38	22,560	−	−	−	−
PI 65	m	27	43,089	−	−	−	−

+ = positive, − = negative, +/− = weak positive.

* This sample contained only gametocytes.

### *Pfhrp2:* percentage of samples with gene deletions and geographic origin

**Figure 2:**
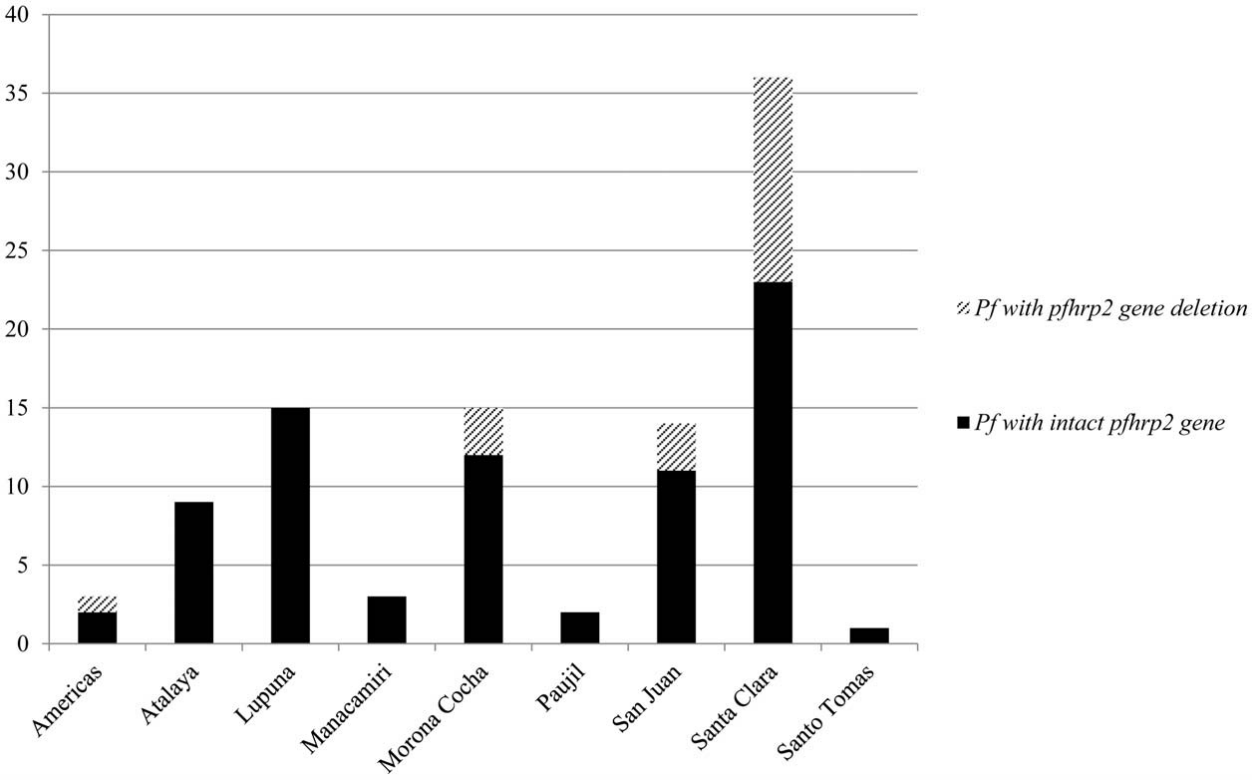
Number of *P. falciparum* samples containing or lacking the *pfhrp2* gene per health center. The village of Atalaya is not a health center, but is displayed separately as all samples in Atalaya were collected by an outreach team during an epidemic.

*Pfhrp2* gene deletions occurred among 19 (25.7%) *P. falciparum* samples. Thirteen (68.4%) were obtained from patients presenting at the health center of Santa Clara (Figure 2), with most patients living in Tarapoto (8/13, 61.5%). The remaining six were distributed among three other health centers (Figure 2). *Pfhrp2* gene deletions were found throughout the study period and sometimes *P. falciparum* samples with and without *pfhrp2* gene deletions were found simultaneously in the same village. One patient with a *pfhrp2* gene deletion diagnosed at Morona Cocha had been travelling to Angamos (close to the Brazilian border) during the month previous to sampling.

### *Pfhrp3* gene deletion

In total 34 (43.6%) *P. falciparum* samples lacked the *pfhrp3* gene: they included all samples lacking the *pfhrp2* gene (n = 19) as well as 15 additional samples which contained *pfhrp2*, and which were correctly diagnosed by all PfHRP2-detecting RDTs.

### Occurrence of cross reactions

In most (10/12) RDT products that were assessed with *P. vivax* samples, *P. falciparum* test lines (either PfHRP2 or Pf-pLDH) were visible at a median frequency of 2.5% (range 1.0%–10.9%). In total, 27 (26.7%) *P. vivax* samples were involved. In all of these samples, mixed infection with *P. falciparum* was excluded by PCR and none of the patients had reported *P. falciparum* infection in the month prior to sampling. In six of these samples however, HRP2 ELISA yielded a weak positive result. There was no apparent relation between parasite density and the occurrence of cross-reactions (range 255–58,880/µl).

In two RDT products, *P. falciparum* samples generated a visible Pv-pLDH line: one faint line for SDFK80 (parasite density 78,208/µl); and a faint and medium line for Onsite (parasite density 53,333/µl and 3,480/µl). Mixed infection with *P. vivax* was excluded by PCR and none of the patients reported recent *P. vivax* infection.

### Interobserver agreement

For positive/negative readings, median κ per RDT product was 1.00 (range 0.84–1.00). For line intensity readings, median κ was 0.87 (range 0.62–0.99).

## Discussion

The present study evaluated a panel of RDT products for malaria diagnosis in the Peruvian Amazon. It showed that Pf-pLDH-detecting RDTs performed significantly better for *P. falciparum* diagnosis compared to PfHRP2-detecting RDTs in this geographical region. The low sensitivity of PfHRP2-detecting RDTs was related to *pfhrp2* gene deletions which invariably leaded to false negative PfHRP2 results irrespective of the parasite density. For *P. vivax* diagnosis all but two RDT products performed well with no overall difference in sensitivity and line intensity between Pv-pLDH and pan-pLDH detecting RDTs. Cross-reactions with the *P. falciparum* line were observed in 10/12 *P. vivax*-detecting RDT products at a median frequency of 2.5% (range 1.0%–10.9%) of *P. vivax* samples assessed. In two RDT products, false positive Pv-pLDH lines were observed in up to 2.7% of *P. falciparum* samples.

### Impact of *pfhrp2* gene deletions

The exact incidence of *pfhrp2* gene deletions in the Peruvian Amazon is not known. We presently found 25.7% of *P. falciparum* samples lacking *pfhrp2*, in a previous study this was 41.0% [8]. In the present study *pfhrp2* gene deletions were found at different sites, but not at all health centers. *Pfhrp2* gene deletions have however been reported throughout the Peruvian Amazon [8] as well as in Brazil [19] and one of the presently included patients might have acquired infection near the Brazilian border. By consequence, the findings as currently described may be applicable to the whole Amazon region.

The impact of *pfhrp2* gene deletions is further highlighted by the fact that all samples lacking *pfhrp2* were not detected by any of the PfHRP2-detecting RDT products. In addition, all samples lacking *pfhrp2* were found in symptomatic patients and occurred at both high and low parasite densities, in contrast to a previous study [20] which demonstrated *pfhrp2* gene deletions only in asymptomatic patients and at low parasite densities. Of note is that in 1998–1999 an evaluation of the PfHRP2-detecting RDT Parasight-F around Iquitos showed sensitivity for *P. falciparum* diagnosis of 95% [9]. Possibly, *pfhrp2* gene deletions have become common in this area only recently.

### Discordances between *pfhrp2* PCR and PfHRP2 RDT results

For samples PI151 and PI156, the presence of *pfhrp2* exon2 was demonstrated by both PCRs but PfHRP2 RDT and ELISA results were negative. Parasite density of both samples was far above the RDT detection threshold and does not explain failure of detection. A mutation or deletion may have occurred, leading to failure of production of the antigen or production of an antigen that is not recognized. Failure of detection of both samples may also be due to errors in transcription or translation, causing low parasite protein expression and consequently failure of detection by RDTs and ELISA [21]. Further research is needed to investigate the occurrence and cause of this phenomenon.

### Role of *pfhrp3*

It has been postulated that PfHRP3 might compensate for absence of PfHRP2 in PfHRP2-detecting diagnosis, due to cross-reaction of PfHRP3 with PfHRP2 antibodies [8], [17]. In the present study this could not be assessed since all *pfhrp2* negative samples lacked the *pfhrp3* gene as well.

### Cross-reactions

In all samples showing cross-reactions, mixed infections were excluded and *Plasmodium* infection during the month previous to sampling was not reported. In the case of *P. vivax* samples generating a PfHRP2 line, past subclinical infection with PfHRP2 persistence (caused by slow clearance of PfHRP2 [22]) may have occurred in at least part of the samples, as supported by the weak positive ELISA results in six samples. However, optical density values in these samples were low and PfHRP2 lines were only visible in few RDT products, which makes non-specific reactions a more plausible explanation. In the case of visible Pf-pLDH lines among *P. vivax* samples and Pv-pLDH lines among *P. falciparum* samples, genuine antigen-antibody reactions [23] or non-specific reactions [7] may have occurred. Cross-reactions (false positive *P. falciparum* test lines) among *P. vivax* samples are particularly relevant in RDTs detecting pan-pLDH: in these cases RDT results are interpreted as *P. falciparum* infection and the patient will not be treated with primaquine, which is needed to eradicate the liver stages. Conversely, false positive Pv-pLDH test lines among *P. falciparum* samples indicate mixed *P. falciparum*/*P. vivax* infection, which will lead to unnecessary treatment with primaquine.

### Limitations

The present study did not include *Plasmodium* negative patients, precluding calculation of specificity and positive and negative predictive values. However, it provides relevant data about RDT diagnostic sensitivity and its relation with *pfhrp2* gene deletions, based upon which suitable RDTs can be selected. Further, we included a large panel of simple one-step RDT products but did not include RDTs with a more complex procedure such as the previously evaluated OptiMAL [24]. Not all RDTs could be performed on fresh samples, though samples had been stored for a short period and had not been exposed to repeated freezing and thawing. Besides, no apparent differences were found between RDT results on stored versus fresh samples. Finally, observers of RDT results were not always blinded to microscopy results provided by the health center.

### Which RDT for the Peruvian Amazon?

From the present study it is clear that PfHRP2-detecting RDTs are not suitable for the Peruvian Amazon, due to the high prevalence of *P. falciparum* samples lacking the *pfhrp2* gene which was invariably associated with false negative results. *Pfhrp2* gene deletions occurred at all parasite densities and all patients were symptomatic. The three Pf-pLDH-detecting RDTs - all combining pan-pLDH - performed excellently for *P. falciparum* and *P. vivax* diagnosis. Among one of them however, an unacceptably high proportion of *P. vivax* samples generated cross-reactions with the Pf-pLDH line, impeding its use. For the remaining two, the high number of faint test lines is of concern as especially in field settings faint lines tend to be overlooked or disregarded as negative [25], [26], [27]. Besides, general limitations of Pf-pLDH-detecting RDTs are a lower sensitivity at low parasite densities [7], [12], [14] and less heat stability, although the latter is currently less important than originally described [7], [14] and SDFK40 reports heat stability up to 40°C (Table 1).

Despite the excellent diagnostic accuracy of SDFK40 and Advantage in the present study, prospective field evaluation on all malaria suspected patients is needed to determine positive and negative predictive values and end user performance. In the long term, the development of an RDT targeting both Pf-pLDH and Pv-pLDH could be considered. Such a combination could, besides diagnosing each of both species, also differentiate between *P. falciparum* and mixed *P. falciparum*/*P. vivax* infections, but is not yet commercially available.
